# The Book World of Medicine and Science

**Published:** 1896-10-24

**Authors:** 


### The Book World of Medicine and Science.

A Manual of Botany. By J. Reynolds Green, Sc.D., F.R.S., F.L.S., Professor of Botany to the Pharmaceutical Society of Great Britain. Vol. II. Classification and Physiology. (London: J. and A. Churchill. 1896.)

The second volume of Professor Green's book is before us, and we have no hesitation in commending it to students. The classification of the cryptogams is perhaps rather scantily dealt with, but the flowering plants meet with full recognition. The various natural orders are well illustrated, and details are given as to the uses of the plants, especially of those which yield drugs. This fact will render the work of great use to medical students, who ought to know something, at any rate, as to the source whence so many of their medicines are derived, even though this knowledge no longer "pays" so well in examinations as formerly was the case. The chapters on Physiology are lucid and interesting, and they gain in importance when it is remembered that their author has himself rendered distinguished services to this branch of botany. The book is well got up, and is illustrated by 1,195 figures, many of which are after the beautiful drawings of Kny. It is well fitted to succeed the older, but nevertheless admirable, work of Bently, on which, as we learn from the title page, Professor Green has based his work.

Rheumatism. By T. J. Maclagan, M.D. Second Edition. (London: Adam and Charles Black. 1896. Price 10s. 6d. 320 pages.)

So familiar are we at the present day with the routine treatment of rheumatism with salicin and salicyl compounds that many of us have forgotten the sensational history of the discovery of these the only specifics for this disease. Just twenty years ago the author of the volume under review, by a marvellous example of logical reasoning and inductive inference, predicted that from a tree or plant which flourished in the low-lying damp localities and in the temperate climates, where rheumatism and rheumatic ailments were prevalent, a medicinal principle was to be found which would effect a cure of the condition. "Looking about," says Dr. Maclagan, "for a plant or tree which flourishes under such conditions, that which most naturally presented itself was the willow—the various species of salix. Among the salicaceæ, therefore, a remedy for rheumatism was sought"—and as we now know, a remedy was found. Next, perhaps, to opium and chloroform, the salicyl compounds are the most wonderful of all drugs, and responsible for the relief of more human suffering than almost any other medicament in the pharmacopœia. The discoverer of such a remedy is entitled to be heard on other points of difficulty which beset the whole question of rheumatism, and many of his generalisations on the pathology and ætiology of the condition are hardly less valuable than those on the purely therapeutic aspects. Broadly stated, Dr. Maclagan regards the poison of rheumatism as of miasmatic origin, and as one which is peculiarly liable to attack the motor apparatus, whether muscular, articular, or vascular—the fibrous elements being more particularly susceptible to the poison. Although his arguments are sound and generally convincing, he does not appear to us to explain satisfactorily why in the rheumatic state of infants the fibrous nodules which are apt to appear on the elbow, the scapula, and other situations, which are particularly exempt from the friction and irritation which generally accompany movement, should occur in these situations. The tonsils, as is well known, are favourite seats for rheumatic inflammation, and yet these structures are not concerned in movement, nor are they fibrous in structure.

In the light of therapeutics we are not convinced that Dr. Maclagan has got at the root of all the difficulties in the complete understanding of the peculiar action of salicylic acid—if it is a specific poison for the microbe of rheumatism—it appears to us somewhat paradoxical that "pain" and "temperature" should be the two chief symptoms which it controls—two symptoms which it can equally well cope with in many other conditions. The salicyl compounds are anodynes, and febrifuges certainly, but are they specifics against the germ proper of rheumatism? If so, why are they practically useless in the chronic condition, and againt many other rheumatic manifestations? In this respect, Pouchet's observations are of value. ("Les Nouveaux Remedes," February, 1896.) We have no space here to discuss these and many other equally interesting points of controversy most ably treated of in this classical work on rheumatism. Dr. Maclagan is an authority and a master of the subject, and every word he writes is worth consideration.

Surgical Emergencies. By Paul Swain, F.R.C.S. (London: J. and A. Churchill. Fifth edition, 8vo., 239 pages. With numerous illustrations.)

This little volume, which has already endeared itself to many a student during the troublous times of examination, and later when he is faced with the real difficulties of practice, once again comes before us crowned with the laurels of a new edition. The older editions served their apprenticeship with honour, and now in this, the fifth, we find all that was useful in the old, which time and experience has not replaced by something better. A few additions we do notice, namely, a condensed note on aseptic surgery, its meaning and practice, and in the chapter on the abdomen we notice the innovations which the most up-to-date surgeons practice, together with full description of the Murphy button with illustrations. In planning out such a scheme of the work as Swain's "Surgical Emergencies," one of the most difficult points in its successful accomplishment lies in the adequate balance of the various sections. In ordinary practice the emergencies of parturition far outweigh in the aggregate those from all other sources. In Mr. Swain's book twenty pages are devoted to these, and considering that the various surgical operations such as cephalotripsy, craniotomy, cæsarian section, versions, and the like are included, this cannot be considered an altogether extravagant allowance of space. The chapter on poisoning, though thoroughly useful and to the point, can hardly be regarded as deserving, on its own merits, a chapter to itself in a work on "Surgical Emergencies." Nevertheless, many a man has been grateful for this chapter. The illustrations, which number 180, are for the most part old familiar friends, but they have served and they will serve again, although in the section devoted to the dislocation of joints, we could well dispense with the old forcible method of pulleys—where brevity has been one of the main objects their inclusion seems altogether unnecessary.

